# Why Are Computational Neuroscience and Systems Biology So Separate?

**DOI:** 10.1371/journal.pcbi.1000078

**Published:** 2008-05-30

**Authors:** Erik De Schutter

**Affiliations:** 1 Computational Neuroscience Unit, Okinawa Institute of Science and Technology, Japan; 2 Theoretical Neurobiology, University of Antwerp, Antwerp, Belgium; University College London, United Kingdom

## Abstract

Despite similar computational approaches, there is surprisingly little interaction between the computational neuroscience and the systems biology research communities. In this review I reconstruct the history of the two disciplines and show that this may explain why they grew up apart. The separation is a pity, as both fields can learn quite a bit from each other. Several examples are given, covering sociological, software technical, and methodological aspects. Systems biology is a better organized community which is very effective at sharing resources, while computational neuroscience has more experience in multiscale modeling and the analysis of information processing by biological systems. Finally, I speculate about how the relationship between the two fields may evolve in the near future.

## Introduction

As a computational neuroscientist, I was quite enthusiastic when the systems biology field appeared on the international scientific agenda of the late nineties. Both fields strongly emphasize the use of computational modeling to predict and investigate the properties of biological systems, and I hoped that they would interact closely and strengthen each other. In fact, some of the early initiatives in systems biology were led by computational neuroscientists [1]. Unfortunately this is not what happened: systems biology went its own way and now the two disciplines largely ignore each other. A glimmer of hope is this journal, *PLoS Computational Biology*, which has attracted both in its editorial board and in the papers it publishes a representative mix of both fields. In this review I will explore what are the most likely reasons for the separation between the two disciplines and argue that this is to their detriment, as they have a lot to learn from each other. I will focus more on computational neuroscience, as I know this field best.

## What Are Computational Neuroscience and Systems Biology?

Interestingly, for both fields the exact definition of what they are about and whether they are defined by computational methods is in dispute (e.g., see the respective entries in wikipedia). They can be defined as either fields of study or as a computational paradigm. In the case of computational neuroscience, the term is often used to denote theoretical approaches in neuroscience, focusing on how the brain computes information [2]. Examples are the search for “the neural code” [3], using experimental, analytical, and (to a limited degree) modeling methods, or theoretical analysis of constraints on brain architecture and function [4],[5]. This theoretical approach is closely linked to systems neuroscience [6],[7], which studies neural circuit function, most commonly in awake, behaving intact animals, and has no relation at all to systems biology. A major venue for this community is the Computational and Systems Neuroscience Meeting (http://cosyne.org). Alternatively, computational neuroscience is about the use of computational approaches to investigate the properties of nervous systems at different levels of detail [8]–[10]. Strictly speaking, this implies simulation of numerical models on computers, but usually analytical models are also included (e.g., the material covered in [9]), and experimental verification of models is an important issue [11]. Sometimes this modeling is quite data driven and may involve cycling back and forth between experimental and computational methods [12]. A typical venue is the Computational Neuroscience Meeting (http://www.cnsorg.org/) and user meetings of specific neural simulator packages. Although these two opposing views are often swept under the carpet, and many scientists attend both conferences mentioned, they are reflected in partially separate communities and sometimes lead to heated debate about how the field should be defined.

Similarly, systems biology has also been described in multiple ways. For some it is the integrative study of the interactions between different components of biological systems, and how such interactions give rise to the function and behavior of a system. This approach is, for example, typified by the (Seattle) Institute for Systems Biology (http://www.systemsbiology.org/). For others, it is an approach using theory and computational modeling in close interaction with experimental verification to understand the dynamical behavior of biological systems [13],[14], sometimes also called computational biology. The major meeting in systems biology is the International Conference on Systems Biology (http://www.icsb-2007.org/).

In the rest of this perspective I will not dwell on these distinctions, and will instead emphasize the computational side of both fields.

## Origins of Computational Neuroscience

The lack of interaction between the two disciplines can be most easily understood from an historical perspective. We'll see that important present-day differences originated in the early days of the respective fields.

It is common to trace the origin of computational neuroscience to the mathematical model Alan L. Hodgkin and Andrew F. Huxley [15] developed of the squid giant axon action potential, though one could also argue for the introduction of the integrate-and-fire neuron by Louis Lapicque one century ago [16],[17]. But while neither paper promoted the use of computational methods in neuroscience directly, the Hodgkin and Huxley model remains a cornerstone of the field and is, surprisingly, still extensively used in its original form [18]–[21].

The next big step was the work of Wilfrid Rall, who used mathematical approaches based on cable theory to show that the dendritic arborizations of neurons strongly affect processing of synaptic input [22]–[24]. He pioneered the use of digital computers in neuroscience and developed the discretized version of cable theory, compartmental modeling [24], which forms the basis for some of the most widely used software packages in computational neuroscience (such as GENESIS [25] and NEURON [26]). His contribution is historically interesting for two additional reasons: his conflict with experimental neuroscientists and the attention to the spatial domain.

Before Rall, neurons were assumed to be isopotential and the electrophysiological importance of dendrites was ignored [27]. Modelers removed the spatial dimension and focused only on temporal aspects of the input-output properties, starting with the introduction of the “point unit” by McCulloch and Pitts [28]. Similarly, experimentalists, who just started making intracellular recordings, assumed that current was mostly confined to the soma (e.g., [29]). This set the scene for the famous conflict between John Eccles and Wilfrid Rall [30] about the need to take current flow to dendrites into account when interpreting data recorded in the soma [31]. Not only did this lead to almost a decade of conflict with Eccles, during which period the latter received the Nobel prize together with Hodgkin and Huxley in 1963, but Rall experienced real problems in getting his early work published [30]. In general, the “rather elaborate and sophisticated considerations” [32] introduced by Rall had only a limited impact on the thinking of contemporary neuroscientists. It wasn't until the early seventies that key concepts introduced by Rall, like spatial summation and dendritic attenuation of synaptic input [23],[24], which are now part of core curricula in neuroscience, became commonplace. The general skepticism of experimental neuroscientists toward the validity of theoretical and modeling approaches compared to the experimental method remains a challenge to the field of computational neuroscience. Though the attitude has improved, it will take a long time before theory is taken as seriously in neuroscience as in physics, and even now many experimental neuroscientists expose rather naïve views on the role of theory. One of the reasons why computational neuroscientists showed so little interest in the emerging field of systems biology a decade ago may be that they were more interested in trying to integrate themselves into mainstream neuroscience.

There is no need to describe the further history of the field in detail. Rall's work influenced both mathematicians [33]–[35] and physiologists [36] so that by the mid-seventies some authors started publishing fairly complex single neuron models and using them in neural network modeling [37],[38]. In general these modelers benefited from the fact that neurophysiology has always been a very quantitative science, providing accurate measurements of currents, voltages, spike trains, etc. Theoretical neuroscience also has many fathers, including Donald Hebb [39] and Norbert Wiener [40], followed by Frank Rosenblatt [41] for the machine learning/connectionist branch, and Werner Reichardt [42] for the neural coding branch.

The term computational neuroscience appeared in the second half of the eighties [10]. Many seminal initiatives were started around that time: graduate programs (for example the CNS program at Caltech in 1986), meetings (the Neural Information Processing Systems meeting, http://nips.cc/, in 1987), summer courses (Methods in Computational Neuroscience at Woods Hole in 1988), standard neural simulator software packages like GENESIS and NEURON [43],[44]; and the first textbook appeared [45].

## Origins of Systems Biology

Traditionally it is assumed that systems biology originated in the late nineties. This neglects the fact that Mihajlo D. Mesarović, Ludwig von Bertalanffy, and their colleagues already proposed applying general systems theory to biology in the sixties [46],[47], though with limited impact. Also important was the ground-laying work performed by several mathematical modeling communities, including those in metabolic analysis [48],[49], physical chemistry [50], cardiac physiology [15],[51], and developmental biology [52],[53]. Unfortunately, most of these efforts were rather isolated, with little influence on the research agendas of the respective experimental communities.

The genomics revolution of the nineties [54], closely followed by proteomics [55] and other omics fields, led to a paradigm shift in biology that caused the rediscovery and popularization of systems biology separately and simultaneously by Lee Hood and Hiroaki Kitano in the late nineties [14],[56],[57]. First, technological innovations turned the affected areas into data-driven discovery sciences [56], where complete listings of all the entities of a system (genes, proteins, …) became possible, and, moreover, these listings were shared easily through databases [58]. In other words, an exhaustive, detailed description of the system became not only feasible but was in its overwhelming complexity often the primary data available. This necessitated new, more integrative approaches to analyzing and manipulating the data, for which systems level theory was the best tool. Second, the same innovations made it also much easier to measure biological and chemical properties quantitatively, producing the numbers needed for computational modeling. Importantly, leading biologists like Lee Hood promoted from the beginning the computational approach as an essential tool to investigate the dynamics of the systems studied. The new field of systems biology leveraged and incorporated most of the preceding work in mathematical biology in a short time, with the notable exception of computational neuroscience.

## Difference in Respective Cultures

The separate origins of the two fields, with computational neuroscience clearly being the more “old-fashioned” one, can explain several major differences in their scientific cultures. I will emphasize two: data-driven modeling and community based standards development.

As already mentioned, systems biology mostly operates in a data-rich environment, where the challenge is more to isolate the important from the less important than to infer unknowns. This is very different from the situation in neuroscience where data is usually incomplete and a lot of guesswork is needed. An example to clarify this issue is the different approaches to networks. A very active area in systems biology is the application of graph theory [59] to analyze the topologies of detailed genetic and molecular networks, as this may shed light on the organizing principles governing their dynamics [60]–[62]. While such approaches are also used in neuroscience [63], most of the work on neural networks simulates **randomly** connected networks to investigate their dynamics [64],[65]. This approach may in some cases simplify analysis [66], but in general it is necessitated by a lack of data. For most neural networks, detailed connectivity schemes are unknown and methods to collect the data are still being developed [67],[68]. In general, computational neuroscience lacks the databases necessary for data-driven modeling, and the databases available tend to suffer from a lack of data (with of course the notable exception of the Allen Brain Atlas at http://www.brain-map.org/, which is genomics). The reasons for this deplorable situation have been described elsewhere [69],[70] and include sociological aspects, but also the overall organization of neuroscience research, which is often fragmented, small-scale, and lacks standardized data annotation [71].

Systems biology inherited the large-scale collaborative approaches common in “industrialized” genomics and benefited from the availability of more mature software development tools in the late nineties [72]. This is a real advantage, as many established computational neuroscience software is shackled by legacy code (such as GENESIS [25], at http://www.genesis-sim.org/, and NEURON [26], at http://www.neuron.yale.edu/neuron/). Even worse, large communities, especially in neural network modeling, still use “homegrown” software that is simply not available electronically. Otherwise, the respective software landscapes may seem similar as one can find in either field both open source (the already mentioned GENESIS and NEURON; E-Cell [73] at http://e-cell.org/ecell/) and copyrighted packages (Mcell at http://www.mcell.cnl.salk.edu/ and NeuroConstruct [74] at http://www.physiol.ucl.ac.uk/research/silver_a/neuroConstruct/; Virtual Cell [75] at http://www.vcell.org/). The differences become more obvious if one looks at some of the infrastructure supporting this software development, in particular the development of standards. For example, compare the terminology efforts of the Neuroscience Information Framework (NIF at http://neurogateway.org/catalog/goto.do?page=.terminology) with the Systems Biology Markup Language (SBML [76] at http://www.sbml.org/). The first is an attempt by the neuroinformatics field to set up data annotation standards for neuroscience, and also covers computational neuroscience. Despite the fact that several terminology workshops have been organized since 2004, it is very difficult to find any online information on this project. Moreover, it is unclear how it relates to the NeuroML ([77] at http://www.neuroml.org/) initiative, which is developing common data formats and associated metadata infrastructure for computational neuroscience. Contrast the top-down, secretive approach of NIF terminology with SBML, which has become a de facto standard of systems biology. This standard model description language encapsulates the full domain of biochemical reaction systems in a single mixed pool. An important reason for SBML's success is the bottom-up approach used in its development, involving all stakeholders in active discussions about the standard and making very effective use of Web-based collaboration tools. This community-based development model is of course copied from the open source movement (note the similarities with Linux) and has resulted in a much wider acceptance of SBML than the competing standard (CellML [78] at http://www.cellml.org/), which is based at a single institution. The success of SBML should be a model for (computational) neuroscience in how to develop standards in a cheap and effective manner, but such a change will not come easy as it goes against the current insular culture of the field.

Of course SBML can also be improved; i.e., despite its wide implementation, few software packages support all of SBML and many limit SBML support to write-only mode. For application in neuroscience, the current version lacks provisions for defining geometry or spatial coordinates, which are necessary to simulate biochemical models of synapses that include detailed 3-D geometry at the submicroscopic level [79]–[81], but this should be solved in the next version.

SBML has not prevented a proliferation of software programs executing very similar tasks (http://www.sbml.org/SBML_Software_Guide), exactly like what happened in computational neuroscience [82],[83]. But the use of SBML strongly enhances portability of models between different programs and therefore promotes sharing and reuse of models through deposition in the BioModels Database (http://www.ebi.ac.uk/biomodels/). An interesting reuse has been to simulate hundreds of models to validate and benchmark simulators (http://www.biouml.org/biomodels.shtml). The situation is very different in computational neuroscience where only now, more than 20 years after their origin, the interoperability between two major neural simulators is being implemented [74],[84] and network simulation packages are being benchmarked [82]. As a consequence, sharing of neural models is still limited and not obligatory, though the model database ModelDB (http://senselab.med.yale.edu/modeldb/) [85] is gaining impact. But almost every computational neuroscientist will have experienced the frustration of trying to recode a model from the literature first-hand. Systems biology therefore serves as an example of how a scientific community can implement standards in a universally accepted manner and how to enforce publishing of model code and scripts.

## What Has Computational Neuroscience To Offer?

There are many areas where systems biology and computational neuroscience have achieved comparable levels of expertise, i.e., in automated parameter searching [86],[87]. But because it is a more mature field, computational neuroscience has more extensive experience in some specific topics [88].

A major strength of computational neuroscience is the accumulated knowhow in simulator software development, especially for multiscale modeling. The latter started when the cable equation introduced by Rall [24] was combined with Hodgkin-Huxley type models [15] of voltage-gated channels to model the effect of neural excitability on synaptic integration [37],[38],[89],[90], but now extends from molecules to large neural networks. To support these simulations, both very specialized and general purpose simulators have been developed. For example, the multiscale simulator GENESIS allows us to include detailed biochemical pathways simulations, using the kinetikit module [91] (http://www.ncbs.res.in/˜bhalla/kkit/), into morphologically detailed neuron models [92] or large neural network models [93].

An interesting specialized simulator is Mcell [79],[80], which should be of great interest to the systems biology community. This mature and well-supported program is highly optimized to simulate reaction–diffusion systems in reconstructed 3D environments. Based on ray-tracing methods, it was first applied to extracellular diffusion and interaction with membrane bound receptors, in particular in the synaptic cleft [94], but Mcell3 now allows simulation of intracellular reaction–diffusion systems in great detail [79]. As already pointed out above, Mcell is not compatible with SBML version 2.

In other cases the interest may not be so much to apply the simulator itself, but to learn from the technical software expertise developed in building it. For example, the NEST simulator ([95] at http://www.nest-initiative.org/) was created to model very large neural networks consisting of fairly simple neuron models. The NEST developers have achieved a deep understanding of distributed event modeling, leading to very efficient, supralinear parallelization of their algorithms [96]. Similarly, it can be expected that the software development done by the Blue Brain project (http://bluebrain.epfl.ch/), which aims to build an extremely detailed tissue model of a cortical column containing tens of thousands of complex neuron models [97],[98], may be of interest to systems biologists.

A challenge common to both fields is how to understand information processing by biological systems. While genetics has progressed from the simplistic view of the genome as a map to more complex gene networks, the types of analysis used do not focus much on the information content. Both genetic and molecular networks are most commonly analyzed using dynamical systems [99]–[101] or graph theory [60]–[62]. This is in contrast to the sophisticated tools used by theoretical and computational neuroscientists to study information processing by neural systems. They have analyzed neural coding at multiple levels of detail, from synapses, over spike trains in single neurons to information processing at the network and at the systems levels [2], [3], [102]–[104]. The methods used consider the neural system as a black box that generates an input to output transform, an approach not commonly used in systems biology. These tools allow for accurate measurement and comparison of information transfer rates using information theoretic analysis [3],[105], detailed characterization of optimal spatiotemporal input profiles using reverse correlation methods [106] and independent component analysis [107],[108], and for definition of optimal coding schemes using Bayesian methods [109] and infomax learning [110]. It is only recently that information theoretic methods have started to enter the systems biology domain [111],[112]. One problem is that such methods often require extensive manipulation of the input space, which may be difficult to obtain in many biological experiments, but this can be overcome by realistic modeling. For example, Mcell modeling has been used to predict binding of attractant molecules to cell receptors, which was then analyzed using information theoretic tools to investigate what would be an optimal coding scheme for chemotaxis [111]. In conclusion, the extensive experience gained in studying neural coding principles may inspire new methods to tackle the high dimensional information processing problems encountered in almost every biological system.

## Looking Into the Future

How do we progress from here? Ideally one would want to promote stronger interaction between the two fields and increased awareness of each other's strengths, i.e., by organizing joint meetings. But at present it seems there is, exceptions like this journal notwithstanding, little interest in the respective communities for such initiatives. In part, this reflects different interests, e.g., system biologists often see computational neuroscience work as too specialized, while many computational neuroscientists have little interests in genes and molecules.

However, I do not believe that the current situation will persist. Faced with the big disparity in funding levels, and even the abolishment of neuroscience specific programs like the Human Brain Project [113], there will be increasing pressure on the computational neuroscientists who would fit most easily into the systems biology world, those modeling at the subcellular and cellular levels, to cross over. Maybe this is already happening. Organizers of meetings like the CNS meeting and of specialized computational neuroscience summer courses (http://www.neuroinf.org/courses/ and http://www.irp.oist.jp/ocnc/) have been noticing a decrease in participants interested in cellular modeling and a shift toward networks and information coding. This has often been attributed to the increased use of cellular modeling techniques by experimental neurophysiologists [114], who are less motivated to attend these events. But an alternative hypothesis is that this reflects a shift of young scientists interested in cellular modeling toward systems biology. If this interpretation is correct, the field of computational neuroscience as we know it will gradually disappear. The more theoretical part, concerned with cognitive operations and the neural code, may then merge further into systems neuroscience, while the bottom-up modelers will become systems biologists. This would not be a very satisfactory outcome, as it will still leave a lot of scientists hanging in between, like bottom-up modelers who want to study cognitive phenomena (see the Blue Brain project).

To prevent this outcome, the field of computational neuroscience will have to explicitly reach out to systems biology and to adapt to some of its conventions, as mentioned before. Eventually, it could then form a bridge between systems biology and neuroscience. A related question, of course, is the stability of the neuroscience field itself and whether it will become a data-driven discovery science [56],[115]. But even if neuroscience would change to such a degree, its strong emphasis on the understanding of human cognition will keep neuroscience distinct from the rest of biology.
